# The Effects of Vitamin D Supplementation on Lipid and Inflammatory Profile of Healthy Adolescent Boys: A Randomized Controlled Trial

**DOI:** 10.3390/nu12051213

**Published:** 2020-04-25

**Authors:** Amirhossein Yarparvar, Ibrahim Elmadfa, Abolghasem Djazayery, Zahra Abdollahi, Forouzan Salehi, Ramin Heshmat

**Affiliations:** 1 School of Nutrition, University of Vienna, 1090 Vienna, Austria; ibrahim.elmadfa@univie.ac.at; 2 Health and Nutrition Specialist, Health and Nutrition Department for UNICEF Regional Office for Europe and Central Asia, Almaty 050000, Kazakhstan; 3 School of Nutrition and Dietetics, Tehran University of Medical Science, Tehran 14155/6117, Iran; jazaiers@tums.ac.ir; 4 Nutrition Department of the Ministry of health and Medical Education, Tehran 1467664961, Iran; abdollahi_z@yahoo.com; 5 Deputy Director of Family Health Department of the Ministry of Health and Medical Education, Tehran 1467664961, Iran; salehi46@yahoo.com; 6 Ramin Heshmat, Tehran University of Medical Sciences, Tehran 141675395, Iran; rheshmat@tums.ac.ir

**Keywords:** vitamin D supplementation, adolescent, inflammation, lipid profile, PTH

## Abstract

Background: Deficiency of vitamin D, an anti-inflammatory micronutrient with some favorable effects on lipid profiles, has been found to be highly prevalent in adolescents. We aimed to investigate the effect of a school-based vitamin D supplementation regimen on the correction of vitamin D deficiency as well as lipid and inflammatory profiles of healthy adolescent boys. Methods: In this randomized single-blind placebo-controlled trial, seventy-one healthy adolescent boys (age 17 years old) were recruited from one high school in Tehran, Iran, and randomly assigned to two groups. The supplement group received vitamin D pearls at a dose of 50,000 IU monthly for 6 months, this dose is indeed defined by the Ministry of Health in Iran for a potential national school-based vitamin D supplementation program. The other group was given placebo pearls for the same duration. Before and after the treatment, the serum levels of 25-hydroxy vitamin D (25(OH) D), parathyroid hormone (PTH), retinol, lead (Pb), the lipid profile and the inflammatory biomarkers were measured and compared. Results: Between-groups statistical analysis showed that a dose (50,000 IU/month) vitamin D significantly increased the serum levels of 25-hydroxyvitamin D (25 (OH) D) (*p* < 0.001) and decreased serum levels of PTH (*p* = 0.003). No significant change was observed in serum levels of retinol and Pb. Between-group analysis revealed that the serum levels of TG (P = 0.001) decreased while an increase in serum levels of HDL (*p* = 0.021) was observed (*p *< 0.05). Both the within- and between-group analysis showed that serum tumor necrosis factor receptor 2 (TNFR2) concentration declined while serum interleukin-10 (IL-10) increased in response to vitamin D supplementation (*p *< 0.05). Conclusion: A supplementation regimen of (50,000 IU/month) vitamin D in a context with high rates of vitamin deficiency has shown positive impacts on the serum vitamin D, lipid profile and inflammatory biomarkers in healthy adolescent boys.

## 1. Introduction

Vitamin D deficiency has become a major public health challenge in many societies. According to the US Institute of Medicine, vitamin D deficiency is defined as serum 25(OH)D levels <30 nmol/L (<12 ng/mL), while vitamin D insufficiency is defined as serum 25(OH)D levels ranging from 30 to 49.9 nmol/L (12–19.9 ng/mL) [1]. The importance of vitamin D for hormonal activity in the homeostasis of calcium, phosphorus and bone metabolism, as well as regulation of immune system, is well acknowledged [2,3,4]. Since, during adolescence, vitamin D requirements increase for the growth of muscles and bones, vitamin D deficiency is often observed during this period [5]. High prevalence of severe vitamin D deficiency among adolescents has been reported in the Middle Eastern and Southeast Asian regions [6,7]. Numerous studies in Iran have highlighted a high prevalence of vitamin D deficiency among adolescents [8,9]. According to the very recent National Integrated Micronutrient Survey-ll (NIMS-ll), 76% of the adolescents in Iran are suffering from vitamin D insufficiency or deficiency [10]. 

Studies have suggested that vitamin D deficiency may contribute to higher levels of inflammation and dyslipidemia [11,12,13]. Longitudinal studies have demonstrated that dyslipidemia and inflammation during childhood and adolescence often persists into adulthood and is associated with atherosclerosis, hypertension, diabetes and cardiovascular disease [14]. It has been suggested that the effect of vitamin D on lipid profiles and inflammation may be mediated through a reduction of parathyroid hormone (PTH) concentration, and via increasing the calcium absorption [15,16].

A national population-based study by Kelishadi et al. in 1095 Iranian students found significant inverse associations of 25(OH) D with total cholesterol (TC) and low-density lipoprotein (LDL) and a positive association with high-density lipoprotein (HDL) [17]. However, interventional studies are necessary to establish the clinical impact of vitamin D supplementation on adolescents. So far, the results from clinical trials have been inconsistent [18,19,20] or mainly focused on adolescents with certain diseases. 

Based on the national evidence presented above, the Ministry of Health in Iran has adopted a national policy and program for school-based vitamin D supplementation (50,000 IU/month) for high school girls all over the country. Before the expansion of the model to high school boys, the Ministry demanded evidence on potential effectiveness of the same regimen on vitamin D, lipid and the inflammatory profile of high school boys.

Therefore, the aim of this study was to investigate the effect of the same dose of vitamin D supplementation on serum levels of vitamin D, blood lipid profile and inflammatory biomarkers on healthy adolescent boys aged 17 years from Tehran in a context with high rates of vitamin D deficiency, with the hypothesis that a regimen of vitamin D supplementation can improve the vitamin D status and both the lipid and inflammatory profile in healthy adolescents.

## 2. Subjects and Methods

**Figure 1:**
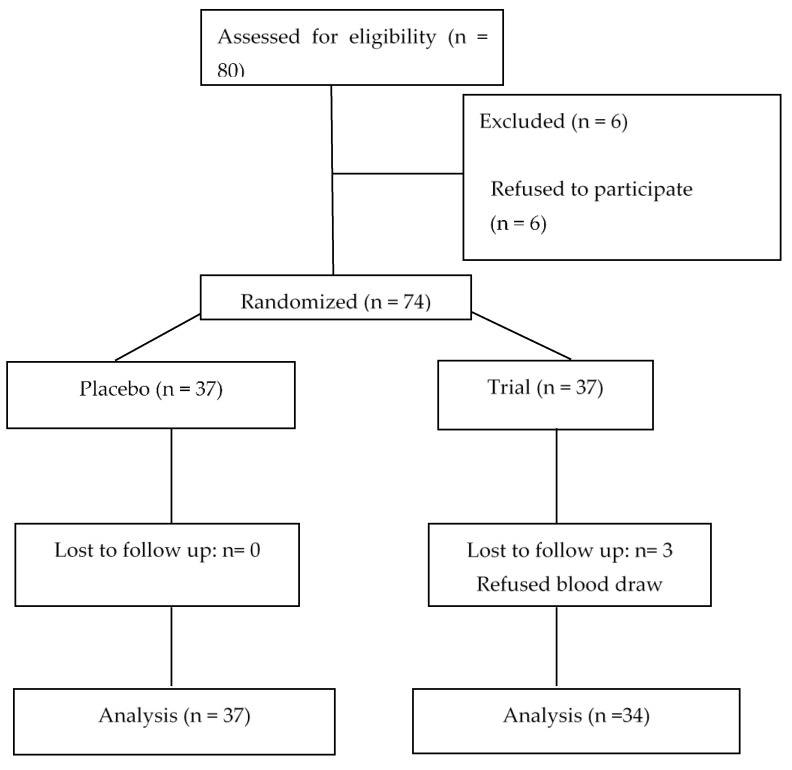
The study flow chart.

This randomized placebo-controlled clinical trial was conducted from September 2018 to May 2019 and included 71 high school adolescent boys aged 17 years from Tehran, Iran. The study was a single blind placebo-controlled trial, as the participants in the trial group were informed if they were receiving vitamin D supplements, and this was inevitable due to the ethics of the national program. To calculate the sample size and based on the reviewed literature in order to have enough samples to compare the two means of serum 25(OH)D before and after the supplementation, with the study power of 80%, α = 0.05, Δ = −8.2, β = 0.95 and σ1 = 40.96 and σ2 = 70.56, the sample size for each group was defined as 31. In order to minimize the risk of drop outs, the sample size in each group had been increased to 37, and ultimately the total sample size for the two groups was 74. Sampling was done in one school, located in district 19 of Tehran. The students were randomly allocated to study groups using simple random sampling and a table of random numbers. The study group decided to opt for sampling from one school firstly in order to be able to minimize the socio-economical differences as well as the dietary and sun exposure patterns. In addition, given the massive and unexpected devaluation of local currency in Iran that took place at that time, the financial power of the research related to the purchase of laboratory supplies was restricted and, hence, the study design needed to be more cost effective. The study protocol has been registered at IRCT (Iranian Register of Clinical Trials; (IRCT20150826023776N4)) and has been considered and approved by the medical ethics committee of Tehran University of Medical Science (NO: IR.TUMS.VCR.REC.1396.4577). Written informed consent was obtained from all participants’ parents. Exclusion criteria included severe renal, liver, lung, cardiovascular, metabolic disease and malabsorption; in addition, students with special diet or supplementation regimes were also excluded. The supplement group received vitamin D pearls at a dose of 50,000 IU administered monthly for 6 months, and the placebo group received placebo pearls for the same duration, both administered by the research team on monthly visits (Figure 1). The vitamin D as well as the placebo pearls were manufactured by Zahravi Pharmaceutical Co., Tehran, Iran. The effects of this supplementation on study subjects were assessed after six months of supplementation. The participants were requested to report any change in drug and supplements use and disease status during the trial. The three day 24 h recall method using a questionnaire was used to collect the dietary data. The individuals were interviewed before and after the intervention about their food and beverage consumption during the previous day, photos were used for defining the portion sizes.

## 3. Laboratory Tests

After a 12-h overnight fast, 10–11 mL of venous blood was obtained from each participant (at 0 and 180 days). The 25(OH) D and PTH of serum were measured using an automated Roche electro-chemiluminescence system (Roche Diagnostics GmbH, Mannheim, Germany). The serum Calcium was measured using the Bromocersol Green Method (Parsazmoon, Tehran, Iran), and Serum Albumin was measured using Arsenazo III (Parsazmoon, Tehran, Iran). The HPLC YL9100 (Young Lin Bldg., Korea) was used to estimate retinol (vitamin A) in serum. The concentrations of serum triglyceride (TG), TC, HDL and LDL were measured by enzymatic colorimetric techniques using commercial kits (Parsazmoon, Tehran, Iran). Serum High sensitivity C-Reactive Protein (hs-CRP) level was assessed using a commercially available immunoturbidimetric method (Biochemical Autoanalyzer BT-1500, Biotecnica Instruments S.p.A, Rome, Italy). Serum levels of TG and HDL were categorized based on the Expert Panel on Integrated Guidelines for Cardiovascular Health and Risk Reduction in Children and Adolescents (2011) [21]. Circulating levels of interleukin-6 (IL-6), interleukin-10 (IL-10) and the soluble form of tumor necrosis factor receptor 2 (TNFR2) were determined by ELISA (Bioassay technology lab, China). The blood levels of lead (Pb) were measured using the Younglin AAS 8020 (Young Lin Bldg., Korea) atomic absorption spectrophotometer by graphite furnace method [22].

## 4. Statistical Analyses

The analyses were performed using SPSS 19.0 (SPSS Statistics for Windows, Version 19.0. IBM Corp., Armonk, NY). All data are presented as mean ± standard deviation. Based on the central limit theorem [23] and the results of the Kolmogorov–Smirnov test, the data distribution was considered normal. Statistical comparisons between groups were done using the Analysis of covariance (ANCOVA), Independent Sample *t* test and Chi-Square test. Comparisons within groups were performed by paired sample *t* test and sign test. *p*-value < 0.05 was statistically significant.

## 5. Results

The study flowchart is given in Figure 1. The general characteristics of the study are presented in Table 1. 

**Figure 2:**
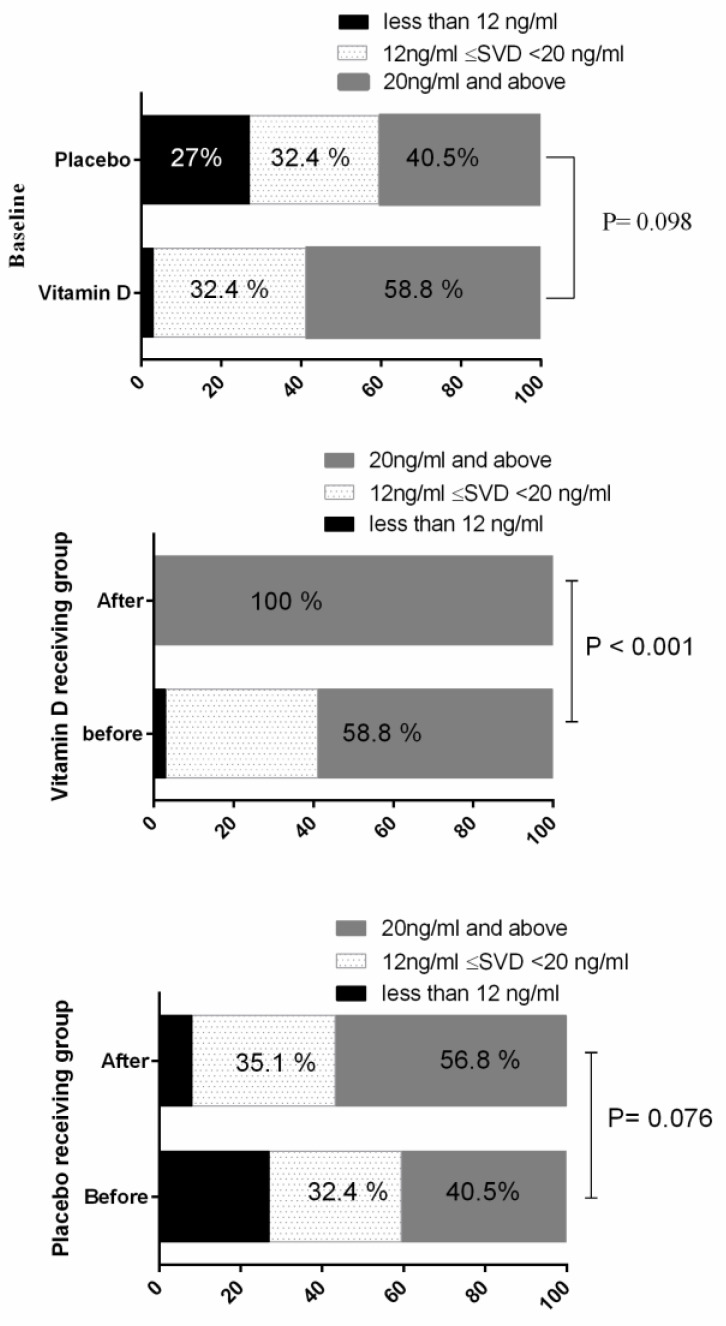
Vitamin D status in the study groups at the baseline and during the study period. Statistical analysis was done by Chi-Square and sign test. Data are presented is percentages. SVD: Serum Vitamin D.

From the study subjects, 59.5 and 41.2 percent of placebo and vitamin D receiving groups had serum vitamin D less than 20 ng/mL at the baseline, respectively. At the baseline, the difference of vitamin D status (less than 12 ng/mL, 12–20 ng/mL, 20 ng/mL and above) between the study groups was not statistically significant (*p* > 0.05). After vitamin D supplementation, serum vitamin D levels in all of the vitamin D receiving subjects were in the normal range (Figure 2).

A total of 71 male students completed the study. Three subjects in the control group refused to continue the trial. In the current study, no side effects were observed and no significant changes in the secondary outcomes such as dietary energy, carbohydrate, fat, vitamin D and vitamin A intake were observed during the study period (Table 2).

Other secondary outcomes were serum levels of vitamin D and A, Pb and PTH, which within-group statistical analysis showed that supplementation with vitamin D significantly increased serum levels of vitamin D (*p* <  0.001) and decreased PTH (P  =  PTH03), while no significant change occurred in the placebo group. No statistical difference was observed on the serum calcium levels adjusted for Albumin levels among the two groups throughout the intervention (*p* = 0.788). On the other hand, no significant change on the serum levels of vitamin A and total blood levels of Pb were observed after the intervention (Table 3). The blood Pb was measured to assess the potential impact of this vitamin D supplementation regimen on bone resorption, which could result in elevation of blood Pb. In this study lipid profile and inflammatory markers were considered as primary outcomes. As shown in Table 4, the parameters of the lipid profile did not significantly change in the study groups during the study period (*p* > 0.005), whereas between-group analysis revealed that the serum levels of TG decreased (*p* = 0.001), while an increase in serum levels of HDL was observed (*p* = 0.021) in comparison with the placebo. Both the within- and between-group statistical analysis showed that serum TNFR2 concentration decreased while serum IL-10 increased in response to vitamin D supplementation (*p* < 0.05). As shown in Table 5, during the study period, serum levels of IL-10 (ng/mL) reached to 109.46 ± 31.78 from 111.11 ± 26.84 (*p* > 0.05) and to 124.22 ± 26.62 from 118.85 ± 26.83 (*p* < 0.05) in placebo and vitamin D receiving groups, respectively. Evaluation of marginal mean differences showed serum levels of IL-10 increased by 6.56 ng/mL in the vitamin D group compared controls and TNFR-2 decreased by 1.64 (ng/L) in the treatment-receiving group (*p* < 0.05).

As shown in Table 4, based on the within-group analysis, the TC and LDL showed favorable significant reduction in the trial group, and the between-group statistical analysis revealed that intervention with vitamin D decreased serum levels of TG and increased serum levels of HDL, respectively, in comparison with the placebo (*p* < 0.05). As shown in Table 5, the inter-group analysis has shown that this supplementation regimen in the trial group has significantly reduced the serum level of IL-6 and TNFR2 and has increased the levels of IL-10 compared to the control group (*p* < 0.05). No impact on hs-CR *p* was observed (*p* > 0.05). 

**Figure 3:**
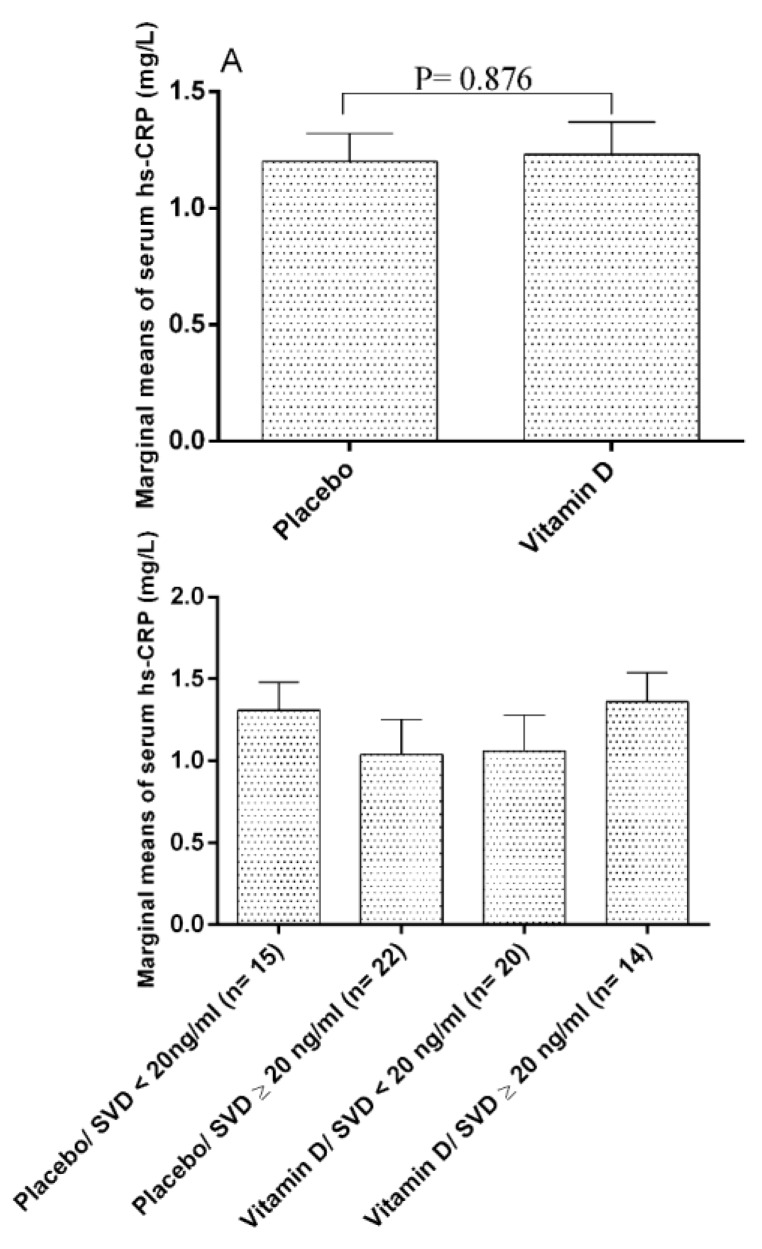
Estimated marginal means of the serum levels of hs-CR *p* in the study groups. Data are presented as Estimated Marginal Means ± SE. data analysis was done by ANCOVA after adjustment of baseline values. hs-CRP: high sensitive C-reactive protein; SVD: Serum Vitamin D. Estimated marginal mean: Post-intervention values after adjusting for baseline values between the study groups.

**Figure 4:**
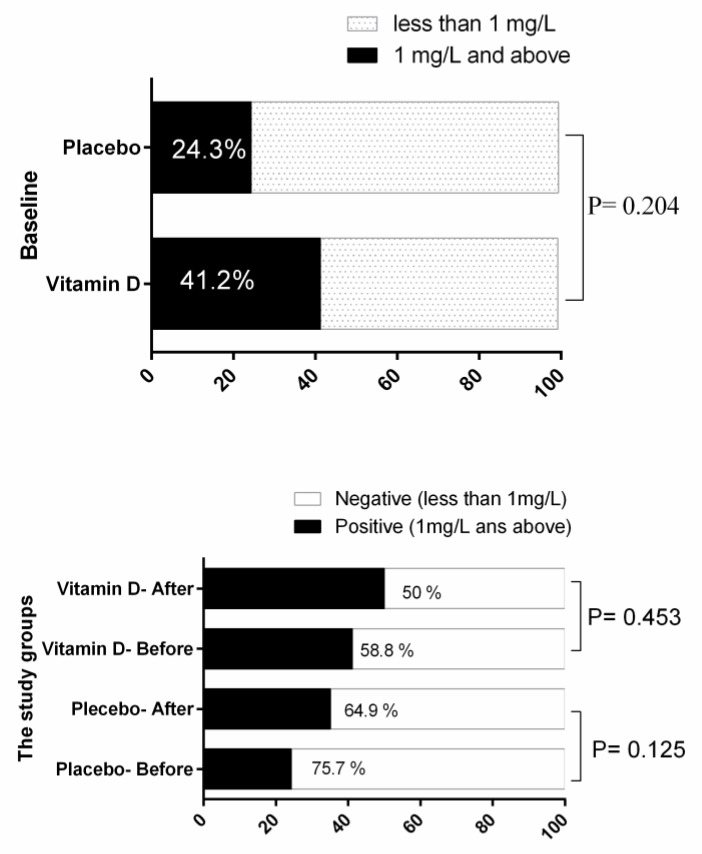
hs-CR *p* status in the study groups at the baseline and during the study period. Statistical analysis was done by Chi-Square and sign test. Data are presented as percentages. hs-CRP: high sensitive C-reactive protein.

The status of serum levels of hs-CR *p* in the study groups is presented in Figure 3. The estimated marginal mean of hs-CR *p* in the placebo group was 1.20 ± 0.12 and in vitamin D receiving group was 1.23 ± 0.14, and the difference was not statistically significant (*p* = 0.876). As it is illustrated in Figure 4, after considering the sub-groups of the vitamin D status, the differences were not significant (*p* > 0.05). Also, qualitative analysis of hs-CR *p* status at the baseline and during the study period revealed no significant effects of the intervention on the hs-CR *p* status (Figure 4).

## 6. Discussion

This randomized controlled placebo trial was aimed to assess the effectiveness of monthly supplementation of vitamin D with a dose of 50,000 IU per month for a period of 6 months, on the serum vitamin D stats, lipid and inflammatory profile of high school students in Tehran. The findings of the current study have shown that this supplementation regimen has resulted in a significant improvement of the serum vitamin D in the trial group compared to the placebo group. Although the administered dose of vitamin D in this study is much less than the recommended does for treatment of VDD (50,000 IU per week), the monthly does of 50,000 IU has significantly corrected the VDD after 6 months of supplementation. 

The supplementation regimen also significantly increased the serum HDL and IL-10 (as an anti-inflammatory interleukin), and a significant reduction in Serum TG and TNFR2 levels was observed. There has also been a significant inter-group reduction of Total Cholesterol and LDL in the trial group, although it was not significant in intra-group comparison; however, the reduction level can be considered scientifically important and favorable. Although intervention studies have suggested that vitamin D supplementation is able to provide the highest beneficial effects in subjects with a severe vitamin D deficiency at baseline [24,25], in our study, due to the low sample size of sub-groups (vitamin D deficient, insufficient and sufficient groups), the sub-group analysis was not scientifically possible and beneficial.

The inverse association of vitamin D and inflammatory markers has been reported by some cross-sectional studies. Amer et al. studied more than 15,000 male and female subjects from the National Health and Nutrition Examination Survey (NHANES) [26]. The study population was divided into groups with low (less than 21.2 ng/mL) and high (21.2 ng/mL and higher) vitamin D concentration. The study results showed that serum levels of vitamin D were inversely associated with CR *p* in groups with a serum 25-OH D lower than 53 ng/mL (53 nmol/L) (β = − 0.34; *p* < 0.001), while in groups with higher vitamin D levels, the mentioned association was not observed (β = − 0.05; *p* = 0.07). Belenchia et al., in a Randomized Controlled Trial (RCT), evaluated the effects of 6 months oral administration of vitamin D at a dose of 4000 IU/day on inflammatory markers in obese adolescents with vitamin D insufficiency, and the results revealed that the intervention insignificantly improved inflammatory markers including IL-6, TNF-α and hs-CR *p* [27]. Moreover, significant effects of interventions with vitamin D on inflammatory markers have been repeatedly reported in adults [28].

It seems that the relationship between inflammation and vitamin D status is reciprocal. Some researchers reported that inflammation reduces vitamin D concentration, while others showed that increasing vitamin D status reduces inflammation, and both seems to be right [29,30,31]. Inflammation decreases vitamin D concentration via oxidative stress induction. Oxidative stress activates vitamin D catabolizing enzymes and disturbs the biosynthesis of the vitamin in the liver. On the other hand, vitamin D itself has the potential to decrease inflammation [28]. Both in vitro and experimental studies indicate that vitamin D and its analogs have potent anti-inflammatory properties [31,32]. Several mechanisms were reported for anti-inflammatory properties of vitamin D and its analogs. Both innate and adaptive immune systems are affected by vitamin D. The overall effect is a shift from a T-helper 1/Th17 (more inflammatory) response to a T-helper 2/Treg (less inflammatory) response. The mechanism results in a smaller production of pro-inflammatory factors such as TNF-α, interferon- γ (IFN-γ) and IL-21, and a higher production of anti-inflammatory markers such as IL-10 [31,33,34]. Cytosolic domain TNFR-2 releases into the general circulation after proteolysis. TNFR-2 acts as a reservoir for TNF-α and prolongs inflammatory effects of the cytokine [35]. It should be noted that, although TNFR-2 has pro-inflammatory properties, it also elicits strong anti-inflammatory activities on oligodendrocytes, cardiomyocytes and keratinocytes [36].

Associations of vitamin D status with the lipid profile have been reported over the last 20 years [37,38,39]; however, very few human studies have been conducted to evaluate the impact of vitamin D supplementation on such indicators on healthy adolescent boys. Wang et al., in a cross sectional study, reported the inverse association of serum vitamin D with TG and LDL in healthy subjects [37]. On the other hand, Tavakoli et al. showed that vitamin D supplementation significantly increased HDL in high school students [19]. Several mechanisms such as a higher activity of lipoprotein lipase [40], regulation of calcium metabolism [41,42] and lower PTH activity [43] have been suggested for the association of vitamin D with the lipid profile. In our study, vitamin D supplementation appeared to have beneficial effects on reducing serum total cholesterol, LDL and, more strongly, TG, as well as effects on increasing the HDL levels in healthy adolescents. Considering the facts that dyslipidemia [44] and vitamin D deficiency are both common health problems among Iranian children and adolescents in Iran, these results are of high potential clinical significance to help reduce the potential risks of cardiovascular diseases. 

Among the limitations we faced, we can refer to the relatively small sample size, which was mainly imposed due to massive and unexpected devaluation of the slocal currency in Iran that reduced the financial power of the research related to the purchase of laboratory supplies. This factor also limited the possibility of sub-group analysis to compare the effectiveness of the vitamin supplementation in vitamin D deficient, insufficient and sufficient subjects. The research team also decided not to include girls into the study design mainly because all high school female students in Iran have been receiving (50,000 IU) of vitamin D supplements on monthly basis through a national program in all high schools. We also did not assess the patterns of sun exposure in the study population; however, given the relatively narrow time span for data collection, the narrow age range and data collection from one school, we did not expect major differences among samples. 

## 7. Conclusions

To our knowledge, this study was the first to investigate the effects of this dose of vitamin D supplementation on inflammatory factors and lipid profile in healthy adolescents. The results of the current study showed anti-inflammatory effects of vitamin D supplementation and lipid improving effects in healthy male adolescents. Considering the high prevalence of vitamin D deficiency in Iran, monthly oral intake of vitamin D with dose of 50,000 IU could be beneficial and recommended for both the correction of vitamin D deficiency and also to improve the inflammatory and lipid profile of adolescent boys. Inflammatory status and dyslipidemia are two main risk factors for cardiovascular disease. Many environmental factors positively or negatively affect inflammation and lipid profile, and vitamin D supplementation as an environmental element could have profound effects on these risk factors in Iran, due to the high prevalence of vitamin D.

## Figures and Tables

**Table 1:** The general characteristics of the study subjects.

Variable			The Study Groups		*p*-Value
			Placebo (*n* = 37)	Vitamin D (*n* = 34)	
Anthropometric variables	Height (cm)		163.22 ± 2.97	165.01 ± 3.05	0.167 *
	Weight (kg)		64.02 ± 4.97	63.21 ± 5.11	0.394 *
	BMI (kg/m^2^)		23.43.00 ± 1.64	22.45 ± 1.45	0.680 *
	BMI	Normal	33 (89.18)	28 (82.35)	0.402 **
		Over-weight	4 (10.81)	6 (17.64)	

BMI: Body Mass Index; quantitative data are presented as mean ± SD, qualitative data are presented as frequency (percent); * *p* was reported based on Independent Sample *t* test; ** *p* was reported based on Chi-Square test.

**Table 2:** Comparing the energy and nutrient intakes per day before and after the intervention between study groups.

Variable		The Study Groups		MMD	*p*-Value **
		Placebo (*n* = 37)	Vitamin D (*n* = 34)		
Energy (kcal)	Before	1896.76 ± 151.35	1920.24 ± 194.77	24.66	0.535
	After	1963.35 ± 160.97	1989.62 ± 169.90		
	PC	4.26	4.40		
	*p*-value *	0.098	0.078		
CHO (gr)	Before	241.04 ± 37.33	251.29 ± 46.99	11.94	0.339
	After	251.22 ± 40.19	256.93 ± 63.62		
	PC	6.69	8.34		
	*p*-value *	0.262	0.201		
Fat (gr)	Before	79.67 ± 14.42	79.69 ± 19.20	−4.89	0.264
	After	83.96 ± 16.41	79.06 ± 20.33		
	PC	9.46	7.10		
	*p*-value *	0.236	0.908		
Protein (gr)	Before	59.27 ± 12.36	57.27 ± 13.17	4.07	0.284
	After	57.62 ± 15.61	61.80 ± 15.88		
	PC	2.43	12.26		
	*p*-value *	0.646	0.183		
Vitamin D (µg)	Before	0.35 ± 0.24	0.36 ± 0.24	−0.59	0.366
	After	0.46 ± 0.27	0.40 ± 0.27		
	PC	136.70	99.48		
	*p*-value *	0.071	0.531		
Vitamin A (µg)	Before	615.73 ± 556.07	625.55 ± 473.41	−105.30	0.267
	After	560.77 ± 450.42	456.49 ± 329.51		
	PC	97.42	2.48		
	*p*-value *	0.610	0.087		

* *p*-value was reported based on Paired sample *t* test. ** *p*-value was reported based on Analysis of Variance (ANCOVA) after adjustment of baseline values. CHO: carbohydrate; MMD: Marginal Mean Difference; PC: Percent Change; PB: Lead; PTH: Parathyroid hormone.

**Table 3:** Comparing the serum levels of vitamin D, vitamin A, PB and PTH in the study groups before and after intervention.

Variable		The Study Groups		MMD	*p*-Value **
		Placebo (*n* = 37)	Vitamin D (*n* = 34)		
Vitamin D (ng/mL)	Before	22.15 ± 12.33	24.16 ± 10.16	17.08	<0.001
	After	23.45 ± 10.10	41.65 ± 6.82		
	PC	16.51	97.66		
	*p*-value *	0.275	<0.001		
Vitamin A (mg/l)	Before	0.485 ± 0.099	0.464 ± 0.072	−0.006	0.731
	After	0.484 ± 0.069	0.470 ± 0.081		
	PC	2.78	2.45		
	*p*-value *	0.913	0.677		
PB (µg/dL)	Before	1.54 ± 1.77	1.25 ± 1.24	0.012	0.953
	After	1.17 ± 0.746	1.13 ± 0.995		
	PC	90.25	114.52		
	*p*-value *	0.133	0.661		
PTH (pg/mL)	Before	25.44 ± 7.42	26.80 ± 7.94	−5.47	0.003
	After	28.59 ± 9.53	23.43 ± 4.91		
	PC	20.70	−8.37		
	*p*-value *	0.111	0.004		
Calcium(mg/dl)	Before	10.80 ± 0.380	10.74 ± 0.400	−0.021	0.788
	After	10.63 ± 0.358	10.59 ± 0.314		
	PC	−1.47	−1.26		
	*p*-value *	0.017	0.054		
Albumin (g/dl) ***	Before	5.15 ± 0.308	5.22 ± 0.322	−0.001	0.992
	After	5.26 ± 0.314	5.29 ± 0.297		
	PC	2.17	1.40		
	*p*-value *	0.071	0.265		

* *p*-value was reported based on Paired sample *t* test. ** *p*-value was reported based on ANCOVA after adjustment of baseline values. MMD: Marginal Mean Difference; PC: Percent Change; PB: Lead; PTH: Parathyroid hormone. *** In order to have an accurate analysis on impacts of supplementation on serum calcium, the serum albumin was also measured and analyzed. The result has shown no significant difference in serum levels of Albumin between the two study groups (*p* = 0.992).

**Table 4:** Comparing the lipid profile in study groups before and after intervention.

Variable		The Study Groups		MMD	*p*-Value **
		Placebo (*n* = 37)	Vitamin D (*n* = 34)		
VLDL (mg/dl)	Before	25.67 ± 12.28	22.94 ± 9.28	−4.75	0.095
	After	29.00 ± 15.33	23.02 ± 8.85		
	PC	18.83	13.81		
	*p*-value *	0.154	0.969		
LDL (mg/dl)	Before	87.83 ± 19.08	84.97 ± 17.62	−5.17	0.092
	After	86.86 ± 20.17	79.44 ± 17.92		
	PC	0.06	−6.24		
	*p*-value *	0.707	0.003		
TG (mg/dl)	Before	130.67 ± 60.21	115.26 ± 45.89	−30.31	0.001
	After	136.34 ± 54.67	99.26 ± 29.30		
	PC	9.52	−1.02		
	*p*-value *	0.383	0.108		
HDL (mg/dl)	Before	43.48 ± 7.79	44.32 ± 8.75	2.93	0.021
	After	42.10 ± 5.89	45.58 ± 8.85		
	PC	−2.06	4.34		
	*p*-value *	0.116	0.282		
TC (mg/dl)	Before	169.62 ± 32.17	164.08 ± 23.31	−6.32	0.213
	After	163.78 ± 32.14	153.47 ± 25.68		
	PC	−2.39	−6.18		
	*p*-value *	0.171	0.002		

* *p*-value was reported based on Paired sample *t* test. ** *p*-value was reported based on ANCOVA after adjustment of baseline values. MMD: Marginal Mean Difference; PC: Percent Change; VLDL: Very Low Density Lipoprotein; LDL: Low Density Lipoprotein; TG: Triglyceride; HDL: High Density Lipoprotein; TC: Total Cholesterol.

**Table 5:** Comparing the inflammatory markers in study groups before and after intervention.

		The Study Groups		MMD	*p*-Value **
		Placebo (*n* = 37)	Vitamin D (*n* = 34)		
IL−6 (ng/L)	Before	129.40 ± 49.60	118.29 ± 40.19	−10.44	0.083
	After	133.64 ± 63.04	112.30 ± 32.75		
	PC	4.22	−3.34		
	*p*-value *	0.425	0.008		
IL−10 (ng/L)	Before	111.11 ± 26.84	118.85 ± 26.83	6.56	0.001
	After	109.46 ± 31.78	124.22 ± 26.62		
	PC	−2.22	4.75		
	*p*-value *	0.332	<0.001		
TNFR−2 (ng/L)	Before	18.37 ± 1.33	19.36 ± 5.21	−1.64	0.019
	After	16.78 ± 1.63	15.44 ± 4.07		
	PC	−8.01	−17.38		
	*p*-value *	<0.001	<0.001		

* *p*-value was reported based on Paired sample *t* test. ** *p*-value was reported based on ANCOVA after adjustment of baseline values. MMD: Marginal Mean Difference; PC: Percent Change; IL-6: Interleukin 6; IL-10: Interleukin 10; TNF-α: Tumor Necrosis Factor α.
